# Factors Associated with Increased Morbidity and Mortality of Obese and Overweight COVID-19 Patients

**DOI:** 10.3390/biology9090280

**Published:** 2020-09-09

**Authors:** Amany Magdy Beshbishy, Helal F. Hetta, Diaa E. Hussein, Abdullah A. Saati, Christian C. Uba, Nallely Rivero-Perez, Adrian Zaragoza-Bastida, Muhammad Ajmal Shah, Tapan Behl, Gaber El-Saber Batiha

**Affiliations:** 1 National Research Center for Protozoan Diseases, Obihiro University of Agriculture and Veterinary Medicine, Nishi 2-13, Inada-cho, Obihiro, Hokkaido 080-8555, Japan; 2 Department of Medical Microbiology and Immunology, Faculty of Medicine, Assiut University, Assiut 71515, Egypt; 3 Department of Internal Medicine, University of Cincinnati College of Medicine, Cincinnati, OH 45267-0595, USA; 4 Researcher, Department of Food Hygiene, Agricultural Research Center (ARC), Animal Health Research Institute, Port of Alexandria 26514, Egypt; diaavet@hotmail.com; 5 Department of Community Medicine & Pilgrims Healthcare, Faculty of Medicine, Umm Al-Qura University Makkah, Mecca 24382, Saudi Arabia; aaasaati@uqu.edu.sa; 6 Department of Microbiology, Paul University, Awka, Anambra State PMB 6074, Nigeria; Christian.uba@pauluniversity.edu.ng; 7 Área Académica de Medicina Veterinaria y Zootecnia, Instituto de Ciencias Agropecuaria, Universidad Autónoma del Estado de Hidalgo, Av. Universidad Km 1, Ex-Hda. de Aquetzalpa, Tulancingo 43600, Hgo, Mexico; nallely_rivero@uaeh.edu.mx (N.R.-P.); adrian_zaragoza@uaeh.edu.mx (A.Z.-B.); 8 Department of Pharmacognosy, Faculty of Pharmaceutical Sciences, Government College University, Faisalabad 38000, Pakistan; ajmalshah@gcuf.edu.pk; 9 Chitkara College of Pharmacy, Chitkara University, Punjab 140401, India; tapanbehl31@gmail.com; 10 Department of Pharmacology and Therapeutics, Faculty of Veterinary Medicine, Damanhour University, Damanhour 22511, Egypt

**Keywords:** obesity, COVID-19, coronaviruses, influenza, thrombosis

## Abstract

Overweight and obesity are defined as an unnecessary accumulation of fat, which poses a risk to health. It is a well-identified risk factor for increased mortality due to heightened rates of heart disease, certain cancers, musculoskeletal disorders, and bacterial, protozoan and viral infections. The increasing prevalence of obesity is of concern, as conventional pathogenesis may indeed be increased in obese hosts rather than healthy hosts, especially during this COVID-19 pandemic. COVID-19 is a new disease and we do not have the luxury of cumulative data. Obesity activates the development of gene induced hypoxia and adipogenesis in obese animals. Several factors can influence obesity, for example, stress can increase the body weight by allowing people to consume high amounts of food with a higher propensity to consume palatable food. Obesity is a risk factor for the development of immune-mediated and some inflammatory-mediated diseases, including atherosclerosis and psoriasis, leading to a dampened immune response to infectious agents, leading to weaker post-infection impacts. Moreover, the obese host creates a special microenvironment for disease pathogenesis, marked by persistent low-grade inflammation. Therefore, it is advisable to sustain healthy eating habits by increasing the consumption of various plant-based and low-fat foods to protect our bodies and decrease the risk of infectious diseases, especially COVID-19.

## 1. Introduction 

The World Health Organization (WHO) reports that over 50% of the world’s existing population is obese and overweight, with a growing annual incidence [1]. An estimated number of over 1.9 billion people are overweight and 650 million are obese, with approximately 25 body mass index (BMI) for overweight people and approximately 30 for obese individuals, corresponding to about 45% of adults worldwide [2]. Overweight and obesity are characterized as an unnecessary build-up of fat, which pose a health risk. Obesity and overweight kill more individuals than underweight [3]. This was associated with the intake of high fat and sugar energy-intensive diets and decreased physical activity. Obesity has been well-identified to be a pro-inflammatory condition [4,5]. It should be noted that excess visceral and omental adiposity results in a pro-inflammatory cytokines efflux affecting systemic cellular processes [6,7]. Obese adults have increased circulating pro-inflammatory cytokines with increased leptin and decreased adiponectin production, causing systemic leptin resistance and potential impact on the standard immune profile [8]. Moreover, a few studies have shown a close connection between chronic low-grade inflammation and adipose tissue expansion [9]. Cytokines secreted from adipose tissue change the nature and frequency of immune cells infiltration [10]. The inflammatory state caused by obesity shows systemic consequences for both individual and global public health. It is well-known to increase the death rate due to increased musculoskeletal disorders, heart disease, and some types of cancer [11]. Moreover, studies show that a large number of obese people are likely to develop more virulent viral strains, extend the spread of the virus across the entire population, and eventually increase the overall death rate of an influenza pandemic [12]. Obesity has also been correlated with certain co-morbidities that are no less dangerous than obesity itself, such as type 2 diabetes mellitus, essential hypertension, coronary artery diseases, atherosclerosis, osteoarthritis, and stroke [13]. Sattar et al. [14] noted that obesity enhances multiple cardiovascular risk factors, premature cardiovascular disease development, and adverse cardiorenal effects. In people with diabetes or at higher risk of diabetes, obesity and accumulated ectopic fat often result in impaired insulin resistance and decreased beta-cell function. Both limit the ability of some patients with diabetes to produce a proper metabolic response to immunological problems, leading to an enormous insulin requirement in severe infections [14]. The increasing prevalence of obesity is of concern, as conventional pathogenesis may indeed be increased in obese hosts rather than healthy ones [6]. Reducing the obesity epidemic will not only improve the life quality of those vast numbers of people affected, but also undermine the impact of obesity on infectious diseases [6]. The current review aims to document the factors associated with increased risks for obese and overweight patients contracting COVID-19 that were noted across countries in the pandemic. Additionally, we outline the prevalence and trends of obesity, then review obesogenic mechanisms, genetic factors involved in obesity, including adipogenesis and hypoxia, COVID-19, obesity and COVID-19, and obesity as a risk factor for infection with COVID-19, as well as obesity mechanisms in COVID-19 infections to which obesity increased morbidity and mortality of COVID-19 patients. Finally, we explain how obesity can increase the risk of cardiovascular disease, immune-mediated disease, several bacterial and viral infectious diseases, and how obese hosts may respond while developing effective antivirals and vaccine. Moreover, we explain the nutritional recommendations and guidelines limiting activity levels to tackle and prevent obesity in our communities for the prevention of chronic diseases and increased adverse reactions to viral pandemics. 

## 2. Methods

### 2.1. Protocol 

This systematic review was carried out on the basis of the Statement of Preferred Reporting Items for Systemic Review and Meta-Analysis (PRISMA).

### 2.2. Search Strategy 

In this review article, a comprehensive search was conducted on May 2020 using the following databases: Research gate, PubMed, Web of Science, and Google scholar along with a manual hand for studies published from 2002 to 2020 using keywords as described in Table 1. We removed duplicated papers, then screened the data, ruled out irrelevant work, and then screened the full-text documents. Inclusion criteria includes a number of factors, involving original articles or review articles, as well as work on COVID-19 and obesity. Although some of the exclusion requirements included non-English documents, inadequate methods, and lack of access to the full text.

### 2.3. Data Collection 

Data from each study were obtained in a standardized form, updating citations from the study, baseline features of the subjects included, appropriate intervention, and findings from the study. Study citations included the first author’s name, study title, and year of publication. Meanwhile, each study’s characteristics related to study design characteristics of the patients and location of the study. We specified the extracted comorbidities of cardiovascular disease, hypertension, and dyslipidemia by other systematic reviews and meta-analysis according to the recognized co-morbidities for obesity and COVID-19 [15,16]. 

### 2.4. Quality Evaluation

The abstracts of the articles acquired during the initial search were read by four independent reviewers. All reviewers approved relevant articles and full-text versions of the articles were revised to identify studies that examine diet, the risk of overweight/obesity, and factors associated with increased morbidity and mortality of obese and overweight COVID-19 patients. Data were synthesized with consistent findings, based on at least three different and high-quality studies.

## 3. Obesogenic Mechanism

It is worth noting that obese patients have consistently higher leptin (pro-inflammatory adipokine) levels and lower adiponectin (anti-inflammatory adipokine) levels. This undesirable hormone environment also induces immune response dysregulation and may lead to the pathogenesis of obesity-related complications [17]. In basal conditions, obese patients show a higher level of various pro-inflammatory cytokines, such as interleukin-6 (IL-6), tumor necrosis factor-α (TNF-α), and MCP-1, produced primarily by visceral and subcutaneous adipose tissue causing an innate immune defect [8]. Once an antigen is introduced, chronic inflammation associated with obesity induces decreased activation of the macrophage and blunted pro-inflammatory cytokine development following macrophage stimulation [18]. Lowered macrophage stimulation following antigen presentation, demonstrate poor success of the obese subject’s vaccination [19]. The remarkable obesogenic microenvironment describes the development of antiviral resistance and vaccine escape varieties in obese individuals [19]. Additionally, T and B cell reactions in obese and obese diabetic patients are weakened, resulting in increased susceptibility and postponement of viral infection resolution [20]. Zhang et al. [21] suggested that leptin resistance could be a cofactor of the 2009 A (H1N1) pandemic influenza, as leptin is considered a key regulator of B cell maturation, development, and function. Likewise, obese patients may appear with functional and numerical lymphocytes changes resulting in poor responses of memory T cell and vaccine efficacy [22]. Obesity prevents both antibody and virus-specific CD8 + T cell responses to the seasonal influenza vaccine; again, an obese host’s suboptimal macrophage functionality and maturation that lead to poor vaccine response [6]. Physical inactivity is another important problem among obese subjects. Compared to lean subjects, sedentary or reduced physical activity is indicative of obese patients. Decreased physical activity itself or mediated by insulin resistance negatively affects the immune response to microbial agents at many immune response stages, involving macrophage activation and proinflammatory cytokines inhibition [23,24].

## 4. Genetic Factors Involved in Obesity

### 4.1. Adipogenesis

Obesity-associated gene expression has been modified more in the adenovirus 36 (Ad36)-infected group than in the control one [25]. Kim et al. [26] observed that Ad36 promotes the production of genes related to adipogenesis in the stem cells, raising the adipocytes number by the proliferation of stem cells (hyperplasia). 

### 4.2. Hypoxia

Oxygen, which limits adipocyte hypertrophy, is not enough in swollen adipocytes [9]. Consequently, obese individuals have adipose tissue that is hypoxic and remarkably contains higher lactate levels. Hypoxic adipocytes are also responsible for inflammation in obesity [27]. Consequently, obesity activates the development of genes induced hypoxia such as those encoding nuclear respiratory factor-1, hypoxia-inducible factor-1α, transcription factor 3 (ATF3), and inflammatory adipokines in obese animals [28]. Ad36 chronic inflammation of the adipose tissue induced by infection is associated with increased M1 macrophages and monocyte chemotactic protein (MCP-1) expression in mice [29]. Surprisingly, the Ad36-infected mice’s body weight and epidermal fat deposits did not decrease due to exercise [30]. Furthermore, exercise could not improve the Ad36-seropositive human BMI-Z score (age 12−14 years) relative to the control group score [30]. Alternatively, exercise and Ad36 infection synergistically reduced non-esterified fatty acids, serum glucose, insulin, and cholesterol levels in Ad36-infected mice [30].

## 5. COVID-19

**Figure 1:**
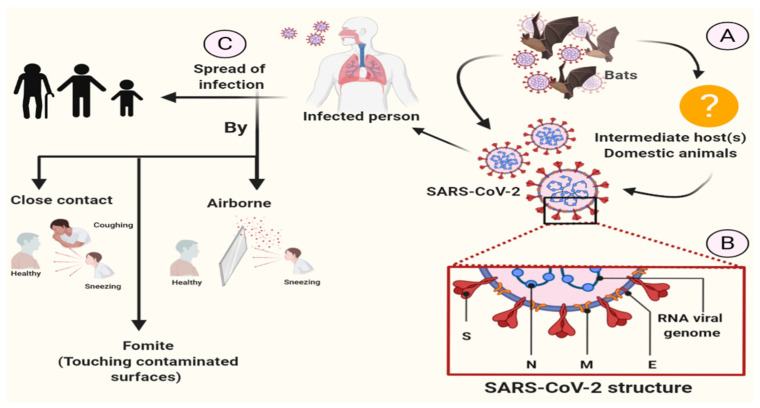
The coronavirus disease (COVID-19) transmission.

The coronavirus disease (COVID-19) pandemic is triggered by the influenza-like strain of the virus (SARS-CoV-2) [15] that causes acute respiratory infection, including a wide range of diseases, from asymptomatic infection to severe acute respiratory damage, in about 20% of patients receiving healthcare [16,31]. The WHO has been notified on 31 December, 2019 that a new virus belongs to the coronavirus family [32]. This infection first developed in Wuhan City and has spread rapidly since December 2019 across China and the world [33]. COVID-19 is a highly transmittable and pathogenic viral infection caused by severe acute respiratory syndrome coronavirus 2 (SARS-CoV-2) (Figure 1). Initial reports suggest that COVID-19 started from the Hunan seafood market at Wuhan, China where bats, snakes, raccoon dogs, palm civets, and other animals are sold and rapidly spread up to 109 countries. Early research has shown that SARS-CoV-2 is primarily spread by direct contact or indirect contact through person-to-person contact, respiratory droplets, contaminated surfaces, and airborne [34]. Although there is an evidence from a single study showing the presence of COVID-19 virus from a single stool specimen [35], no fecal-oral transmission of COVID-19 virus has been reported to date [34]. 

**Figure 2:**
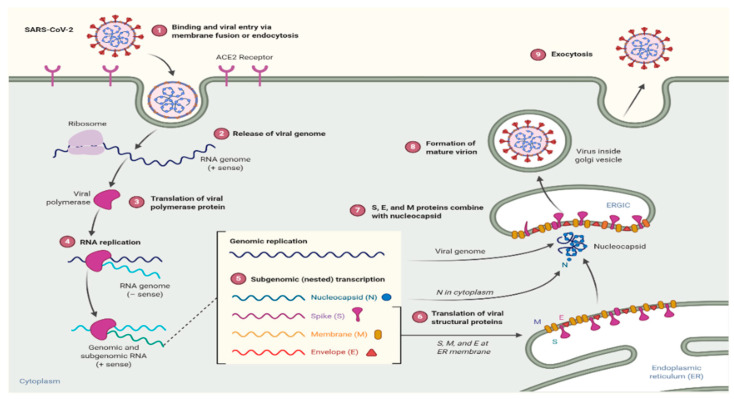
Mechanism of COVID-19 in the host [40].

SARS-CoV-2 enters the human host through binding to angiotensin-converting enzyme 2 (ACE2) found in the lower respiratory tract of humans, the same receptor as with SARS-CoV [36]. Viruses using the two S glycoprotien subunits, S1 and S2, determine the virus-host range and cellular tropism with the key function domain and cause the virus cell membrane fusion by two tandem domains, heptad repeats 1 (HR1) and HR2 [36,37]. Upon membrane fusion, the viral genome RNA (uncoated) is released into the cytoplasm, which translates two polyproteins, pp1a and pp1ab, which encode non-structural proteins, to form a replication-transcription complex (RTC) in a double-membrane vesicle [36,38]. A nested set of subgenomic RNAs that encode accessory proteins that interfere with the host’s innate immune response and structural proteins are produced through continuous RTC replication [38] that will eventually result in a newly formed genomic RNA with endoplasmic reticulum (ER) and Golgi, as well as nucleocapsid proteins and envelope glycoproteins that are assembled to form virions (viral particle buds) [36,39] (Figure 2).

## 6. Obesity and COVID-19

**Figure 3:**
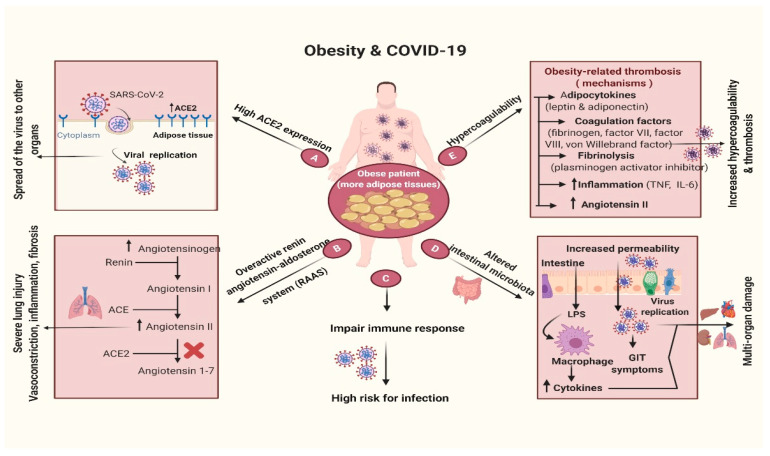
COVID-19 and obesity.

Regrettably, obesity is an unfavorable factor for COVID-19 patients. In this scenario, obesity may lead to more critical signs and complications. The intubation of obese patients is extremely challenging. Diagnostic imaging can be harder to obtain. It is more difficult for the nursing staff to move or place obese individuals and hence the need for special beds and equipment [3]. Currently, newspapers have stated that obesity is an underestimated risk factor for COVID-19 [14,41]. This risk is particularly significant in the United States as the incidence of obese adults (with a BMI of approximately 30) is about 40% compared to 6.2% in China, 20% in Italy, and 24% in Spain [42]. Reported death rates vary geographically, as South Korea, China, and Italy registered a case death rate of 0.8, 2.3, and 7.2, respectively, while citing provincial risk factors, including incidence of population, aging, smoking, or pollution [43,44]. Americans have a high risk of exposure to obesity according to the WHO criteria [45]. Obesity is a newly established epidemiological risk factor in people <60 years of age that can lead to increased morbidity in the United States [44] (Figure 3). 

Simonnet et al. [46] found that COVID-19 patients with grade II obesity had OR: 7.36 (1.63–33.14; *p* = 0.021) times higher risk of in-hospital mechanical ventilation compared to COVID-19 normoweight using a multivariate analysis. This is associated with higher mortality rates in the obese population infected with COVID-19. In addition, Lighter et al. [44] classified their study subjects by age, splitting them into classes of under and over 60 years. In the younger group of patients with obesity grades I and II, the hospitalization rates increased 2.0 (1.6–2.6; *p* < 0.0001) and 2.2 (1.7–2.9; *p* < 0.0001) times, compared with norm-and overweight groups, respectively. The hospitalization rates increased 2.0 (1.6–2.6; *p* < 0.0001) and 2.2 (1.7–2.9; *p* < 0.0001) times in the younger group of patients with obesity grades I and II, respectively compared with normo-and overweight groups. Additionally, younger group patients with obesity grades I and II were more likely to receive critical care 1.8 (1.2–2.7; *p* < 0.006) and 3.6 (2.5–5.3; *p* < 0.0001) times, respectively, compared to normo-and overweight groups. Although Wu et al. [47] recorded an increased risk of 1.30 (1.09–1.54; *p* = 0.003) times in COVID-19 patients with BMI above 25 kg/m^2^ to develop extreme COVID-19 relative to normo-and overweight patients, the multivariate analysis has attenuated this danger.

### 6.1. Obesity as a Risk Factor for Infection with COVID-19

Early high-risk indications for developing serious SARS-CoV-2 symptoms involve hypertension, cardiovascular disease, cancers, and chronic respiratory diseases [47]. For the very first time, Cai et al. [48] identified that the risk of severe pneumonia was enhanced by obesity in COVID-19 patients. Mehra et al. [49] recorded that in an investigation of 96,032 hospitalized COVID-19 patients worldwide, obesity is an independent predictor of higher mortality rates. CDC identified severe obesity (i.e., BMI up to 40 kg/m^2^) as a significant clinical risk factor for worse prognosis and overall death in COVID-19 patients [16]. In addition, certain obesity rates (BMI ≥ 30 kg/m^2^) have been closely related to poor prognosis in COVID-19 patients [50]. Obesity is also linked to impaired pulmonary function, decreased expiratory reserve volume, functional capacity, and respiratory system compliance [51]. Excessive abdominal obesity jeopardizes the pulmonary function in supine patients by reducing diaphragm excursion, while lung ventilation is also compromised, resulting in reduced levels of oxygen-saturated blood [51]. Simonnet et al. [46] identified a risk factor for severity in COVID-19 patients that was solely dependent on the BMI value. Nevertheless, Petersen et al. [52] indicated that visceral adipose tissue and upper abdominal circumference directly raise the risk of COVID-19 incidence and proposed the use of CT-based visceral adipose tissue quantification as a specific risk assessment tool for SARS-CoV-2 patients on regularly obtained chest CTs. Certain factors that exacerbate the clinical evolution of COVID-19 infection in obese patients are the difficulty of pulmonary ventilation in these subjects, with reduced diaphragmatic excursions and a remarkable increase in anatomical death space [12].

### 6.2. Obesity Mechanisms in COVID-19 Infections

Obesity demonstrates insulin resistance and an overactivity of the renin angiotensin-aldosterone system (RAAS) related with bad outcomes in COVID-19 [53]. As a result, SARS-CoV-2 can also contaminate the adipose tissue and spread to other organs [54]. In addition, obesity is the major cause of type 2 diabetes mellitus (T2DM) together with a low PA/fitness, and T2DM is also causally associated with a high angiotensin-converting enzyme (ACE2) expression [55]. Moreover, cells that express ACE2 are associated with developing idiopathic pulmonary fibrosis (IPF) [55]. Zheng et al. [56] revealed that obesity could exacerbate sever acute coronavirus-2 (SARS-CoV-2) respiratory syndrome (SARS-CoV-2). Regarding the movement of COVID-19 epicenters towards Europe and North America, the impact of obesity on COVID-19 may become much more evident, as these two regions have some of the world’s highest rates of obesity taking on epidemic proportions [51].

## 7. Quarantine and COVID-19

Hâncu and Mihălţan [15] hypothesized that the quarantine era may be characterized as an “obesogenic” era, where sedentarism is preferred, whereas there is increased propensity to consume hypercaloric foods, which are consumed unconsciously due to stress. Abbas et al. [3] asserted that with the continuation of the COVID-19 pandemic, there will be a decline in dietary supplements with an increased tendency for food storage and increased use of canned food and ultra-processed items because of their safe processing and packaging, as well as the probability of eating in their other activities. This substantially contributes to an increase in the global obesity burden, particularly with reduced exercise and home-stay measures. This may be described by: (a) High sugar, salt, and fat content, (b) high calorie content that may surpass the calorie intake of an adult, (c) high processed carbohydrate content that may contribute to a change in the insulin response and brain reward system, resulting in excess food concentration in the adipose tissue, as well as addictive-like behaviors [57,58].

## 8. Key Gaps in Knowledge to Direct Future Research 

We review the peer-reviewed and pre-printed literature on COVID-19-related obesity considerations and highlight key information gaps that need further research specific to patients, health care workers, and health systems. Obesity was rarely reported in early clinical studies assessing the clinical risk factors for COVID-19 disease. Few data from a single-center retrospective research stated that obesity seems to have a high prevalence between COVID-19 patients admitted to intensive care needing intrusive mechanical ventilation and the disease severity increased with BMI [59]. For instance, a recently published study on a large number of COVID-19 patients younger than 60 years linked higher BMI values with an increased likelihood of acceptance into intensive care [44]. Therefore, immediate studies are needed to reduce mental health problems and, in particular, to promote well-being in vulnerable individuals. A coordination mechanism for pandemic mental health interventions is needed for identifying the intervention gaps that will need tailor-made de novo design, evaluating and rolling out remotely delivered interventions. High-quality data are required to determine over time the impact of lockdown and social isolation, with a rising risk of obesity. Innovative work is required to find ways to reduce and control mental health risks [60]. Particularly targeted epidemiological studies are needed to demonstrate the obesity effect on the incidence and mortality rates of COVID-19, and hence to establish effective therapeutic strategies for obese patients.

## 9. Obesity and Stress

Some individuals may become depressed, stressed, and unwilling to exercise, while others will consume more food without any action as a result of COVID-19 spreading negative news, which can lead to weight gain and become obese [61]. Yet, it also exerts pressure on psychological well-being by fear of virus infection, home treatment, social isolation, financial distress, speculations, and lack of information [3]. All of these will result in higher levels of stress and anxiety, which may lead to more physical health problems, such as obesity. A recent study demonstrated the association between severe obesity and stress, increased energy intake, and reduced diet quality [62]. Stress can influence body weight via behavioral, biological, and psychological pathways. Stimulation of the hypothalamic-adrenal-pituitary axis, can lead to the release of cortisol that may influence body weight by stimulating eating alone, reducing brain sensitivity to Leptin, and stimulating the reward pathway [58]. Stimulating brain reward centers such as dorsal striatum and nucleus accumbens may increase the propensity to eat highly palatable foods containing high amounts of sugar, fat, and sodium [62]. Stress has an impact on the brain regions responsible for self-regulation that is necessary for regulating one’s habits, such as eating a meal and physical exercise, which are vital for weight management, decreases the desire to exercise, and allows people to consume higher levels of food with a greater tendency to eat palatable food [3].

## 10. Gut Microbiome and Obesity

Gastrointestinal symptoms identified in SARS-CoV-2 infection, such as abdominal distension, diarrhea, and bloating, may be dramatically improved by restoring microbiota equilibrium. A micro-ecological treatment can minimize the translocation of bacteria, increase positive bacteria, and decrease toxin production. Infection due to intestinal bacterial dysbiosis might be prevented. Gut biota analysis seems not to be affordable and thus not available as a routine test, but dysbiosis could be defined on the basis of symptomatology. Probiotics can be effective in the prevention or recovery of dysbiosis [63]. Obesity is closely linked to environmental factors such as behavior, exercise, and diet. Scientists are currently studying the correlation between obesity and gut microbiome. Gut microbiota can alter the homeostatic relationship between the gut flora and the intestine and this can lead to metabolic dysfunction [64]. Several studies have documented that the intestinal microbiota modulates metabolic processes in diabetes, NAFLD, and obesity [64]. First, germ-free (GF) rodents were shown to gain less weight than traditional rodents when fed with high sugar and lipid diets [65]. In comparison, the fat mass of GF mice that provided cecal microbiota from ob/ob mice was higher than in mice inoculated with gut bacteria from lean mice [66]. Published studies have shown that obesity reduces the Bacteroidetes abundance and enhances the number of Firmicutes linked with a higher level of carbohydrate degradation and fermentation enzymes that help in efficient dietary energy harvesting [67]. Apart from adipose tissue (AT), obesity impacts the modulation of intestinal microbiota, increasing intestinal permeability, bacteria, and lipopolysaccharides (LPS) translocation to the circulation [68]. These modifications in the intestinal mucosa increase cellular infiltration and amplify the contact between LPS and other pathogen-associated molecular patterns (PAMPs) with toll-like receptors (TLRs), mostly TLR4 [69]. These receptors are known to trigger inflammatory signaling pathways in immune cells, including T cells, dendritic cells, and macrophages [70]. 

## 11. Obesity and Thrombosis in COVID-19 Infections

The frequency of COVID-19 cases is closely related to hypercoagulable condition and thrombosis [71]. Anomalous clotting parameters are related to poor early diagnosis in all individuals with serious coronavirus pneumonia [72]. Overweight and obesity have been continuously associated with a high risk of developing venous thromboembolism (VTE) [73]. Hypercoagulability was observed in overweight patients without metabolic syndrome (MetS) and increased obesity severity [74]. Numerous pathways are associated with obesity-related hypercoagulability and/or thrombosis, including: Hyperactivity coagulation factors (fibrinogen, factor VII, factor VIII, von Willebrand factor), increased inflammation (TNF-α and IL-6), adipocytokines action (e.g., leptin and adiponectin), hypofunctional fibrinolysis (plasminogen activator inhibitor [PAI]), lipid and glucose tolerance disorders along with MetS, raised angiotensin II (endothelial dysfunction and elevated PAI-1), and increased oxidative stress and endothelial dysfunction, as well as venous stasis and compromised venous return [75]. Hence, increased thrombosis and hypercoagulability in patients with COVID-19 may result from the additive effects of obesity and SARS-CoV-2 infection [76]. 

## 12. Obesity and Immunity

Obesity itself is an independent and causal risk factor for the development of immune-mediated disease, e.g., psoriasis, suggesting that such adipose condition may have a systemic immune consequence upon additional environmental provocation. In fact, investigations suggest that obesity interferes with the various pathways of immune system. Some strategies include reduced cytokine production, modified lymphocyte and monocyte function, decreased dendritic cell and macrophage function, natural killer (NK) cell dysfunction, and lowered response to antigen/mitogen stimulation [77]. Obesity was suggested to cause premature immune system aging, reflecting an immunocompromised state, and encouraging both aged and immunocompromised hosts to develop novel viral variants [78]. There is a strong correlation between obesity and basal inflammatory status marked by elevated circulating rates of IL-6 and C-reactive protein. Adipose tissue in obesity is “pro-inflammatory,” with an elevated production of cytokines and adipokines. Dysregulated tissue leukocyte expression is also present, and inflammatory macrophages (and innate lymphoid) replace tissue regulatory (M2) phenotypic cells [14]. An immune disorder is also linked to obesity, with high vulnerability to infection or bacteria [79]. The activity of T-lymphocytes and their subpopulations is disrupted in obese people [80]. 

### 12.1. Obesity and Antiviral Immunity

Obesity leads to a dampened immune response to infectious agents, leading to weaker post-infection impacts [11]. Systemic changes to antiviral immunity, involving adaptive and innate responses, are explained for respiratory epithelium IAV infection [6]. The lung-resident alveolar macrophages number are substantially reduced in OB and DIO mice due to infection, and those remaining cause the IFN type I and IFN-stimulated gene expression to decrease related to WT mice [81]. These alterations could be partially because of the chronic inflammation associated with obesity, as the chronic inflammatory mouse model shows a marked decrease in macrophage activation and blunt pro-inflammatory cytokine production following stimulation of macrophage [18]. In terms of the host defense, obesity affects the adaptive immune response to the influenza virus and is conceivable to do so in COVID-19 [14,82]. Low immunity levels are not acceptable for the protection of a novel coronavirus. It has been noted that 50% of COVID-19-infected individuals are usually diagnosed with hypercytokinemia. Severe cases appear to possess lymphopenia, particularly T cells, leukocytosis and increased neutrophil-lymphocyte ratio (NLR), lower percent of basophils, monocytes and eosinophils, and higher inflammatory cytokines, including TNF-αIL-6, IL-2R, and IL-10. We believed that COVID-19 would have more impact on younger populations than previously reported in populations with a high prevalence of obesity [83]. The same phenomenon may occur with the influenza A virus (IAV) due to blunt immune responses and prolonged viral shedding in obese humans [84,85]. However, Karlsson et al. [86] observed that the delayed interferon response at an early stage of infection allows for increased viral replication and spread, and may lead to increased viral diversity and severe changes were reported in both obese mice and host primary human respiratory cells [87].

Articulating the obesity impact on the immune response to IAV has been achieved through the use of genetically obese (OB) mice missing leptin signaling receptor and diet-induced obese (DIO) models. In DIO mice, IAV-infected mice had a 7-fold higher death rate and pulmonary pathology, and slow wound healing compared to lean controls [85,86]. Likewise, research on IAV infection in OB mice indicates increased disease frequency, increased risk of secondary bacterial infection, reduced vaccine efficacy, and delayed wound healing [19,88,89]. The severity of the enhanced IAV disease could be due to the delayed and blunt immune response, as interferon and adaptive cellular and antibody-mediated responses are all decreased in OB mice [85,86].

Dendritic cells (DCs) promote the immune system’s T-cell response by displaying phagocytosis antigens [90]. Among the human study population, obese patients have decreased the circulating DCs amount and the remaining patients are less responsive to ex vivo stimulation with TLR agonists than non-obese patients, although, more experiments are required to evaluate if this finding applies to IAV infection [91]. Moreover, neutrophils display a pro-inflammatory N1 phenotype in DIO mice, while those from obese humans demonstrate an elevated development of inflammatory free radicals after ex vivo enhancement [8]. Contrarily, NK cells are reduced in all DIO mice, obese and overweight humans, but increased in OB mice [91]. Malnutrition is reported to raise the incidence of IAV disorder and affect NK cell activity in WT mice, while OB and DIO mice display enhanced NK cell infiltration of the lung at days six and fourteen postinfection, while DB mice have demonstrated a decline in NK cells in BALF at day four postinfection [92]. Metabolic dysregulation associated with obesity is identified as a cause of weak T cell effector and T cell helper function and compromised T cell memory and vaccine effectiveness, since it can modify T cell metabolism [93]. 

### 12.2. Obesity and Atherosclerosis

A lot of information about the pathophysiological mechanisms of obesity and atherosclerosis have been discovered over the past three decades. These conditions have historically been considered as lipid storage disorders with accumulation of triglycerides in the fat tissue and cholesterol esters in atherosclerotic plaques [94]. There are many important factors to pathogenesis of obesity and atherosclerosis. In both cases, the inflammatory process is triggered by lipids, oxidized LDL particles, and free fatty acids and cause the disease. Inflammation is responsible for all measures against atherosclerosis, from early endothelial failure to complicating atherosclerotic plaques and is correlated with type 2 diabetes, insulin resistance, and obesity. The fatty tissue releases adipocytokines that cause insulin resistance, endothelial dysfunction, hypercoagulation, and systemic inflammation, thus promoting the atherosclerotic cycle. In the case of visceral obesity, inflammatory adipocytokines (e.g., leptin, TNF-α, MCP-1, resistin, and IL-6) are elevated. In addition, the elevated C-reactive protein level is associated with a higher risk of myocardial infarction, peripheral vascular disease, and diabetes mellitus [95,96]. It is worth noting that a clinical study of obese women has shown that the reduction in body weight achieved by lifestyle changes decreases inflammatory biomarkers and insulin resistance levels [96]. 

### 12.3. Obesity and Psoriasis

Psoriasis is a widespread inflammatory chronic skin disease with a complex pathogenesis composed of an immune dysfunction environmental factor and genetic aspect. Several studies produced evidence that obesity predisposes patients to psoriasis and enhances psoriasis inflammation [97]. Setty et al. [98] suggested that adiposity and weight gain were risk factors for psoriasis development in a study including 78,626 women. They revealed that individuals with a BMI > 35 had a comparatively increased risk of 2.69 psoriasis development compared with lean patients. A recent retrospective research showed that the risk of psoriasis was increased by obesity and high abdominal fat mass, suggesting weight loss, maintaining a normal body weight, and reducing body mass may decrease the risk of psoriasis [99]. Obesity affects the cellular structure and function of inflammatory skin cells. Nakamizo et al. [100] identified IL-17A-producing γδ T cells accumulation in psoriatic skin lesions of high fat diet (HFD)-induced obese mice, which resulted in an exacerbation of psoriatic dermatitis. Moreover, Neimann et al. [101] reported that 3854 persons with severe psoriasis (OR, 1.8) showed a higher risk of obesity than in 127,706 subjects with mild psoriasis (OR, 1.3). Similarly, Cohen et al. [102] revealed that patients under the age of 35 were more likely to be overweight (OR, 2.2) than those above the age of 65 (OR, 1.6) in comparison to healthy individuals. 

## 13. Obesity and Vaccines

Effective preventive strategies and control measures for influenza are important, in particular in the overweight and obese community. This may be problematic due to weak vaccine responses among obese or overweight individuals [89]. Clinical studies will continue to investigate the specific characteristics of the obesogenic microenvironment that facilitate the growth of a more virulent population, because this has an effect on the antigenic drift and for developing antiviral-resistant and vaccine escape variation in obese individuals [20]. With increasing the demands for effective IAV antivirals and vaccines, it is important to consider how the obese host may respond; furthermore, the effects that the obese population may have on potentially growing IAV quasi-species virulence could be much more relevant [103]. Obesity was linked to a high cough and more skipped school days in one study in elementary school children. Vaccination has been shown to be generally safe for both obese and non-obese children [104]. A further investigation on adults vaccinated with a trivalent influenza vaccine observed that a raised BMI was correlated with a substantial decrease in the protective immune response [105]. Honce et al. [87] suggested that the decreased interferon response is partly responsible for the early loss of viral control, enabling the production of virulent IAV strains, as they documented differing rates of interferon and ISG expression in genetically obese mice up to day three post-infection. Although the performance of adjuvant vaccines is better than that of non-adjuvants vaccines when comparing their neutralizing responses, as both vaccines have failed to protect both OB and DIO mice from the homologous viral challenge [19]. Obese individuals fail to induce adequate T-cell and antibody-mediated immunity when infected and vaccinated and release more infectious viruses than lean subjects [106].

## 14. Obesity and Pathogens

Torres et al. [107] concluded that the infection in obese animals and individuals is likely to change according to the spread of infection probably because it affects the metabolic pathways of immune cells in different manners. Observational studies and meta-analyses have linked obesity and infectious diseases in humans. Recently, Dhurandhar et al. [108] have gone over a systematic review of human trials that assessed the effects of obesity on infections and vice-versa. The impact of obesity in H1N1, for example, is important and thus makes obesity a required risk factor in cases of influenza infection [109]. In other several bacterial and viral infections, such as Dengue and HIV, obesity was linked to the worst prognosis in humans [110,111]. There is decreased thymopoiesis and limited T cell receptor diversity in mice with DIO. Yet, peripheral immune response to obesity has reduced the migration of APCs to peripheral lymph nodes and the number of T lymphocytes. These alterations cause imbalances in the leukocyte population distribution and the activity of lymphocyte [11]. Thus, these alterations could affect the immune response against infectious diseases.

### 14.1. Obesity and Respiratory Tract Infections

Frequent and more intense respiratory tract infections (RTIs) have also been a major cause of morbidity in our community and a substantial cost burden in terms of hospital care and working time reduction [112]. Overweight (BMI ≥ 25 kg/m^2^) and, in specific, obesity function (BMI ≥ 30 kg/m^2^) are commonly discussed in relation to RTI vulnerability [113]. Many investigations involving adults have studied the relationship of obesity with different RTIs and their results. Consequently, obesity was found to be linked to non-allergic rhinitis and influenza-like disease [114,115]. Maccioni et al. [116] has shown that there is a relationship between obesity and RTIs that supports previous works on influenza-like ailment, bronchitis, and pneumonia [115,117].

### 14.2. Obesity and Viral Infection

Since the 1918 “Spanish” flu pandemic, it is understood that malnourishment (both under-and over-nutrition) is associated with a poorer viral infection diagnosis [118]. Infection with certain pathogens is well believed to trigger metabolic dysfunction, including diabetes and obesity [26]. In the obese host, there is a growing occurrence of infections and a rise in serious influenza pandemics owing to enhanced viral shedding, dissemination, and the production of novel viral variants [6]. The obese host creates a special microenvironment for illness pathogenesis, marked by a persistent low level of inflammation, which leads to the suppression of innate and adaptive immune responses [11]. Obese individuals may demonstrate greater viral shedding that indicates a potential for high viral exposure, particularly if many family members are overweight [14]. Additionally, the length of the intensive care stay and the need for artificial ventilation have increased due to obesity [119]. Most notably, extreme obesity has contributed to a two-fold rise in the risk of death from IAV infection and hospitalization due to infection, as well as a small increase in the risk of obesity [109]. Additionally, obesity increased the risk of IAV infection in other high-risk individuals, such as pregnant and postpartum women [120]. Few case trials listing obesity as a comorbidity factor increased viral replication and severe hemorrhage in the alveoli resulting in increased severity of the disease [121]. On the other hand, morbid obesity was not a statistically significant risk factor for the Middle East Respiratory Syndrome (MERS) [122]. This might be demonstrated by a relatively high obesity and/or morbid obesity prevalence in both cases and controls which may have obscured the impact of established influenza risk factor and serious acute respiratory syndrome [123]. In addition, severe heavy weight could have limited the movement of patients in an emergency, as they tend to be bedridden most of the time and therefore less susceptible to infection [122].

### 14.3. Obesity and Adenovirus

Avian adenovirus (Ad) (e.g., SMAM-1), a variety of human adenoviruses (e.g., Ad36 and Ad5), and canine distemper virus have been tested for their capacity to induce animal and human adiposity [124]. These viruses increase body weight and fat mass after infection and could be considered a possible risk factor for obesity. In fact, the relationship between obesity and Ad36 is well established in both animals and humans. Ad36 raised the fat content and body weight in infected mice and primates and may retain fat amounts in adipose tissues and in infected animals [125]. Rats infected with Ad36 showed 23% more fat pads than non-infected rats [125]. The latest meta-analysis of clinical trials has demonstrated that Ad36 infection may result in developing obesity or weight gain in humans [25]. Intraperitoneally injection of Ad36 to rats resulted in increased body weight, reduced insulin sensitivity, and increased glucose intake relative to the control group [126]. Rats infected with Ad36 demonstrated that the virus could be transmitted to peripheral organs, as well as liver, adipose tissues, kidneys, brain, and spleen within four days of intranasal infection [125]. The relation between obesity and viral infection is more complicated than a mere deterioration of influenza infection and clinical presentation and results in individuals. Ad36 is the most investigated infectious agent related to obesity. Adenoviruses are DNA viruses primarily responsible for respiratory infections that are shown to increase adiposity in both animal and human models [127]. There are very many mechanisms by which Ad36 infection can cause obesity. First, the increase in chronic inflammation caused by NF-kB activation of the macrophage chemoattractant protein-1 (MCP-1) [29]. Secondly, a drop in the production of leptin in adipocytes contributing to lipid accumulation through a decline in lipolysis, a decline in adipocyte genes modulation included in lipid oxidation, and fatty acid synthesis [128]. Thirdly, the increase in glucose uptake in the adipocytes leads to an energy surplus in glucose-dependent cells [126]. Fourth, adipogenesis upregulation is attributed to an increase in the differentiation and proliferation of confirmed adipogenesis [129]. Sang et al. [130] recently revealed that a high fat diet (HF) and Ad36 reduced immune/inflammatory genes relative to HF alone in the intestine. In particular, HF and Ad36 inhibited interferon gene mechanisms at the transcriptomic level. Such results indicated that Ad36 reduced the antiviral response and high inflammation level to chronic inflammation in the intestine. The virus will conserve energy for immune reaction and cause anabolism and adipogenesis. In general, Ad36 infection can sustain low levels of inflammation in the intestinal and fat tissues and promote the production of lipids to improve the control of blood sugar [26].

### 14.4. Obesity and Influenza Virus

The 1957–1960 “Asian” and the 1968 “Hong Kong” influenzas indicated that diabetes and obesity led to higher death rates and longer periods of disease, as subjects did not have any other medical disorders that raise the likelihood of influenza-related complications [106]. In addition, obesity was associated with a high risk of serious illness and a risk factor for hospitalization and mortality during the 2009 H1N1 pandemic IAV [131]. Obese people are now classified as having an increased risk of severe problems caused by influenza infection [16]. Obesity is closely linked with raising the severity of disease and the viral titers in exhaled breath and substantially extended viral spreading during the influenza A infection [87]. Since the 2009 H1N1 pandemic, epidemiological researches all over the world have noticed obesity in nearly one-third of hospitalized patients and fatal cases to be co-morbid with influenza [132]. Many research studies of serious and fatal IAV infections have revealed the potential influence of obesity on the progression of the disease, including substantial viral replication in the deep lungs, advancement to viral pneumonia, and sustained spreading of the virus [121]. Obesity raises the risk of hospitalization for confirmed laboratory cases of IAV infection during non-pandemic and pandemic influenza seasons, with BMI increasing the odds ratios [133]. In extreme cases, the IAV infection can cause the respiratory epithelium to break down, leading to fluid inflow into the airway [134]. Influenza virus infections are highly heterogeneous, and several small variants of obese donor-passed viruses have been believed to enhance pathogenicity in mice [135]. Although there was no consensus between variants in obese host-passed viruses, there was an increase in the mean genetic diversity of the viral segments. 

#### 14.4.1. Influenza Virus in Obese Mice

Numerous research findings on IAV infection in OB mice indicate a high incidence of disease, elevated secondary bacterial infection, and decreased vaccine effectiveness [136]. In addition, the immune response in obese mice is delayed and blunt, presumably due to decreased interferon (INF), adaptive cellular and antibody-mediated reactions. Honce et al. [87] discovered that serial transmission of human H1N1 influenza virus via OB or DIO mice leads to more virulent IAV population related to a lean host. Viruses transmitted in the obese host have replicated to higher viral titers, resulting in higher morbidity in wild-type (WT) C57BL/6 mice. These observations are restricted to the viral subtype since the transmission of human H3N2 seasonal virus did not demonstrate observable pathogenicity in WT mice. Deep-sequencing viruses obtained from lean and obese-host viruses showed many mutations in obese host-passed viruses primarily concerned with virulence in the mouse model. Moreover, these observations are not limited to the viral subtype, but are also detected in human studies. Decreased INF reaction and enhanced replication of the influenza virus were observed in typical epithelial bronchial (NHBE) cells originating from obese subjects [87,137]. Additionally, high virulence and variability were vulnerable to host interferon responses, because exogenous interferon treatment to OB mice reduced viral variability. Honce et al. [87] ascertained that obesity leads to a specific microenvironment related to impaired interferon response which is permitted to generate more virulent IAV populations and faster adaptation of the viral host. There is a noticeable ATP level and PDC activity downregulation in H1N1 virus-infected mice, limited upregulation of PDK4 in liver, skeletal muscles, lungs, and heart. Influenza is marked by metabolic disorders and a cytokine outbreak. In this case, PDC plays a crucial role in catalyzing pyruvate oxidative decarboxylation and connecting glycolysis to fatty acid and tricarboxylic acid (TCA) synthesis [12]. Yamane et al. [138] showed that oral administration of dichloroacetate (PDK4, DADA inhibitor) in mice retained ATP level and PDC activity, improved metabolism disorders, viral replication, blocked cytokine storm, and trypsin upregulation. Obese mice are often more prone to have decreased lung permeability during infection than lean (LN) mice. Increased lung permeability is associated with increased lung oedema and oxidative stress on IAV infection, underlining numerous etiologies of increased lung pathology in the obese hosts [136]. Increased immunopathology and the slow healing process of wounds in DIO and OB mice lead to high death rates. IAV strains H3N2 and H1N1 cause higher mortality in DIO and OB mice than in WT C57BL/6 mice, irrespective of their vaccine history [19,136]. This is also true of the viral-bacterial co-infection model. DIO and OB mice inoculated with PR8 IAV, CA/09 IAV, seasonal H3N2 virus, or influenza B virus and inoculated with *Streptococcus pneumoniae* at day 7 post-influenza infection increased mortality when compared to controls [89]. Even though some researchers observed higher virus titers in fat mice than in fatty animals, others reported no such difference [139]. In a virus-bacterial co-infection study, the virus load between wild and obese mice (WT) was significantly different from peak disease; however, at later time points obese mice had higher virus titers than WT controls [89]. Obese mice have also increased viral spread to the LRT. More viral antigens were observed in the bronchiolar and alveolar regions of DIO mice inoculated with the H1N1 virus than in similar regions of the infected control animal [88]. Excised lungs from OB mice revealed that increased viral dissemination was present as early as day 3 pi [81]. 

#### 14.4.2. Influenza Virus in Human Cells

Remarkably, this evolution phenotypic expression was preserved using human primary respiratory cells. IAV reproduced to higher titers of normal human bronchial epithelial cells (NHBE) from obese donors than those of lean individuals. Both obese mice and obese-donor NHBEs had reduced interferon and interferon-stimulated genes (ISGs) and developed post-infection, potentially decreasing viral shed control and restricting viral replication [137]. Ritter et al. [140] observed that the effect of influenza viruses on infected mammalian cells typically results in a metabolic transformation, increased glycolytic levels, and reduced ATP synthesis. Thus, they proposed that the initiation of apoptosis triggers this metabolic imbalance during the final stage of the influenza virus replication cycle. Moreover, Wang et al. [141] documented that obese patients diagnosed with serious IAV had higher peak viral loads and prolonged clearance compared to non-obese patients.

### 14.5. Obesity and Spreading of Pathogens

Obese individuals are more susceptible to infection than lean ones because of the following factors. First, obese individuals with influenza spread the virus for a longer period of time (up to 104% prolonged) than slim subjects and significantly raise the risk of transmitting the virus to others [84]. Secondly, the obese microenvironment encourages the appearance of new and more virulent strains of the virus. In addition, that is mainly due to the decreased and delayed production of interferons by obese people and animals [87]. Delays in the production of interferon to contrast viral replication make it possible for more viral RNA replication and this increases the probability of developing novel and more virulent viral strains [87]. Thirdly, the body mass index is closely related to the respiratory infectious virus in exhaled air [142]. This discovery was particularly important for males, which led directly to the assumption that higher volumes of ventilation or differential chest conformation may explain this fact [142]. The enhanced time of viral shedding was unique to influenza A viruses in obese persons, although there was no link between obesity and length of influenza B virus spreading [143].

## 15. Obesity Control

In the current pandemic of COVID-19, physicians should understand that obese, and much more extremely obese, are at greater risk of health decline with COVID-19. Consequently, these patients need to be closely observed and more effectively treated to minimize morbidity and mortality [76]. It is advisable to sustain healthy eating habits by increasing the consumption of various plant-based, low-fat foods, fruits, vegetables, and unprocessed foods with moderate daily exercise at home [144]. Rising numbers of recent studies have strongly associated obesity to more serious COVID-19 disease and death [44,46,50]. Therefore, Sattar et al. [14] proposed that obesity or sustained ectopic fat accumulation may be a common risk factor for acute COVID-19 infection, diminishing both the protective cardio-respiratory reserve and improving immune dysregulation, which tends, at least in part, to mediate progression to serious disease and organ dysfunction in a reasonable proportion of COVID-19 patients. Furthermore, obesity enhances thrombosis, which is important because of the correlation between extreme COVID-19 and pro-thrombotic intravascular coagulation with high venous thromboembolism levels. Obesity has adverse effects on lung function, reduced forced expiratory volume, and forced vital ability beyond cardiometabolic and thrombotic implications [14]. Treatment for people admitted to intensive treatment units is often affected with extreme obesity (e.g., BMI > 40 Kg/m^2^), as these patients are more difficult to image, ventilate, nurse, and rehabilitate [14]. William et al. [145] revealed that the role of vitamin D in lowering the risk of infections has been clarified by cathelicidins and defensins, with reduced activity of the virus replication and lowering proinflammatory cytokines activity. Research supports the suggestion that a dose of 10,000 UI/day of vitD3 should be prescribed for a few weeks for people at risk of developing COVID-19, rather than a preventive dose of 5000 UI/day. The aim must be to sustain a concentration level of 40–60 ng/mL at 25(OH) D. The potential benefits of mass quarantine should be carefully monitored to improve cardiovascular risk over the long term. An unhealthy lifestyle and anxiety should be carefully addressed [15]. This is particularly challenging with current stay-at-home guidelines limiting activity levels—the “lockdown cost of weight gain”. Indeed, this pandemic has demonstrated the need to do more, not less, to tackle and prevent obesity in our communities for the prevention of chronic diseases and increased adverse reactions to viral pandemics [14]. Recently, nutritional recommendations and guidelines have stressed the importance of understanding entire diets and patterns of eating rather than a reductive way that relies on specific foods or nutrients. The Dietary Advisory Committee recommends that overweight and obese individuals achieve weight loss by following a balanced eating pattern [146]. Expert panels concerned both with dietary guidance generally and with the prevention of specific diseases emphasize the importance of obesity prevention [147,148]. Reducing unnecessary disposable calorie intake is a technique that is based on evidence and supported by expert opinion [149]. CDC identified four of the six principal target areas for the prevention and regulation of obesity tackling particular dietary behaviors: Decrease sugar-sweetened beverages, high-calorie, energy-dense foods consumption; increase fruits and vegetables consumption [150]. The evidence base that supports its effect on caloric intake and/or obesity varies for each behavioral target. 

## 16. Obesity and Exercise

Obesity is described by increasing the number and size of adipocytes that promote lipid aggregation and induce a low inflammation level [151]. Exercise and physical activity are strongly correlated with beneficial effects in metabolic (metabolic syndrome, obesity, and diabetes) and immunological (cell response, immunization effectiveness, and cell senescence) fitness. In fact, physical exercise strategies have clearly proven the ability to decrease the risk of complications by modulating inflammation, increasing the immune response, and strengthening vaccine results in elderly people [152]. Many findings indicate that daily physical activity raises the degree of cytokine development induced by TLR (toll-like receptor) signaling pathways during microbial infections, thereby increasing host resistance to pathogen invasion [23]. An additional advantage of exercise is to enhance the antioxidant protecting mechanism and eliminate oxidative stress. Warren et al. [153] showed that exercise enhanced host immunity toward IAV infection in non-obese and obese mice. Specifically, physical exercise in obese mice reversed obesity-related deficiency in the host immune response [153]. These results indicate that physical activity improved immune activation (cell infiltration, chemokine, and bronchoalveolar lavage (BAL) cytokine) earlier throughout infection, restoring the immune response in obese mice to the normal phenotype. Obesity, in fact, has delayed the immune response instead of completely suppressing it [12]. Exercise greatly influences the energy balance and leptin response, muscle PDC activation, type I IFN response, and increases the anti-influenza virus-specific IgG2c antibody in serum and the percentage of CD8+T cell in BAL. All these mechanisms may be considered necessary to protect the host from infection [153]. Zheng et al. [23] investigated that daily exercise improves the immune response to microbial antigens in humans. It is important to understand that beneficial immunomodulation can only be accomplished with mild to moderate frequent exercise. On the other hand, high or extensive exercise is known to suppress the immune response, mainly due to an increase in the endogenous cortisol [154]. Daily physical activity was the basis for the preventive measures needed to improve host susceptibility to influenza virus infections and other metabolic disorders in overweight people. Nevertheless, the rise in overweight and metabolic syndrome among teenagers in the US and other developed countries also represents an additional issue [155].

## 17. Conclusions

Obesity is a medical disorder with complex pathophysiology, containing multiple pathways that now appear as major risk factors for COVID-19. Obese patients have higher leptin (pro-inflammatory adipokine) levels and lower adiponectin (anti-inflammatory adipokine) levels that induce immune response dysregulation and may lead to the pathogenesis of obesity-linked complications [17]. Additionally, they show a higher level of various pro-inflammatory cytokines, such as IL-6, TNF-α, and MCP-1, produced primarily by visceral and subcutaneous adipose tissue causing an innate immunity defect. Stress and anxiety are among several factors that lead to more physical health problems and obesity. Curbing the obesity epidemic will not only improve the quality of life for these affected people, but will also reduce the effect of obesity on infectious disease. In the current pandemic of COVID-19, physicians should understand that obese and overweight individuals are at greater risk of health decline with COVID-19, and therefore, careful observation and more active treatment should be employed to minimize morbidity and mortality in obese patients. Physical exercise strategies have clearly proven the ability to decrease the risk of metabolic syndrome, obesity, and diabetes by modulating inflammation, increasing the immune response and strengthening vaccine results in elderly people. Many findings indicate that daily physical activity raises the degree of cytokine development induced by TLR (toll-like receptor) signaling pathways during microbial infections, thereby increasing host resistance to pathogen invasion. Moreover, it is important to consider how an obese host may respond while developing effective antivirals and vaccine. Therefore, targeted epidemiological studies are needed to demonstrate the obesity effect on the incidence and mortality rates of COVID-19 and other infectious diseases, and hence to establish effective therapeutic strategies for obese patients.

## Figures and Tables

**Table 1:** Search queries of this comprehensive review.

Databases	Search Query
Research Gate	(Obesogenic OR obese OR obesity OR overweight OR BMI) and (SARS-CoV-2 OR COVID-19 OR coronavirus) and (quarantine OR hospital care OR hospitalization OR mortality OR morbidity)
PubMed	(Obesogenic OR obese OR obesity OR overweight OR BMI) and (morbidity OR mortality OR mortality rate OR death rate) and (influenza OR COVID-19 OR coronavirus OR SARS-CoV-2 OR health care)
Web of Science	(Coronavirus OR COVID-19 OR SARS-CoV-2 OR influenza) and (thrombosis vaccine OR WHO OR hospitalization OR intense care) and (obesity OR BMI OR overweight OR care episode OR morbidity OR mortality)
Google Scholar	(SARS-CoV-2 OR COVID-19 OR coronavirus) and (obesogenic OR obese OR obesity OR overweight OR BMI OR stress OR control) and (hospitalization OR hospital stay OR mortality OR morbidity)
Hand searching	Obesity and COVID-19 and immunity
